# Multi-Spectral Food Classification and Caloric Estimation Using Predicted Images

**DOI:** 10.3390/foods13040551

**Published:** 2024-02-11

**Authors:** Ki-Seung Lee

**Affiliations:** Department of Electrical and Electronic Engineering, Konkuk University, 1 Hwayang-dong, Gwangjin-gu, Seoul 143-701, Republic of Korea; kseung@konkuk.ac.kr; Tel.: +82-02-450-3489

**Keywords:** convolutional neural network, multispectral imaging, food recognition, caloric estimation, image conversion

## Abstract

In nutrition science, methods that accomplish continuous recognition of ingested foods with minimal user intervention have great utility. Our recent study showed that using images taken at a variety of wavelengths, including ultraviolet (UV) and near-infrared (NIR) bands, improves the accuracy of food classification and caloric estimation. With this approach, however, analysis time increases as the number of wavelengths increases, and there are practical implementation issues associated with a large number of light sources. To alleviate these problems, we proposed a method that used only standard red-green-blue (RGB) images to achieve performance that approximates the use of multi-wavelength images. This method used RGB images to predict the images at each wavelength (including UV and NIR bands), instead of using the images actually acquired with a camera. Deep neural networks (DNN) were used to predict the images at each wavelength from the RGB images. To validate the effectiveness of the proposed method, feasibility tests were carried out on 101 foods. The experimental results showed maximum recognition rates of 99.45 and 98.24% using the actual and predicted images, respectively. Those rates were significantly higher than using only the RGB images, which returned a recognition rate of only 86.3%. For caloric estimation, the minimum values for mean absolute percentage error (MAPE) were 11.67 and 12.13 when using the actual and predicted images, respectively. These results confirmed that the use of RGB images alone achieves performance that is similar to multi-wavelength imaging techniques.

## 1. Introduction

Understanding the nutritional content of foods consumed is important for the proper treatment of a variety of conditions, which is not limited to problems associated with metabolic diseases such as obesity [1]. To accomplish such an understanding, it is necessary to continuously monitor the type and amount of ingested food. Conventional methods of monitoring the type and amount of food consumed are manual recording methods [2,3,4]. Recently, diet-related apps have been implemented on mobile devices to make it easier for users. The accuracy of this approach, however, is affected by user error and inattention, which makes it less useful.

To partially solve this problem, several types of automatic food recognizers (AFRs) have been developed by which the amounts of and types ingested foods were continuously monitored with minimal user intervention. AFRs are divided into several categories based on what cues are used for classification. A technique that uses sound (acoustics) to identify food types is based on the sounds produced when food is chewed or swallowed [5,6,7,8,9]. Throat microphones [5,6] and in-ear microphone [7,8,9] were used as the acoustic sensors. When applying an HMM-based recognizer to acoustic signals recorded by a throat microphone to classify seven foods, a recognition rate of 81.5∼90.1% was reported [6]. In a food recognition study using an in-ear microphone, it was reported to achieve an accuracy of 66∼79% for seven different foods [8]. However, since it is difficult to distinguish different foods based on acoustic signals alone, there are limits to what can be achieved with acoustic cues.

Since foods have their own unique shapes, textures, and colors, visual cues have been used to classify food types and estimate portion sizes [10,11,12,13,14,15,16,17,18,19,20,21]. From a classical vision-based pattern recognition perspective, automatic food classification is implemented through a series of processes: segmentation, feature selection, and classification of food images. As neural networks have been applied to various image recognition tasks, attempts have been made to use artificial neural networks (ANN) to categorize food types [14,19,20] and estimate the calorie content of foods [14,18,21]. Caloric estimation using visual cues is based on the following assumptions: (1) The calorie counts per size (weight) of a food are uniquely determined by the food item. (2) Food items can be identified primarily by their appearance. (3) The size of a food can be estimated from visual information. When using a convolutional neural network (CNN) to classify foods and predict calories for 15 different food items, it was found to be more accurate than classical pattern recognition [14]. Previous image-based food analysis techniques have primarily used RGB images acquired under visible light. This method has the advantage of easy image acquisition, but it is limited by poor food recognition accuracy for visually similar foods. Another limitation is the inability to utilize the specific response that certain foods emit to ultraviolet and infrared light.

To overcome these limitations, food analysis based on multispectral analysis has been widely adopted [22,23,24,25,26,27,28,29,30,31,32,33,34,35,36,37]. The basic principle is that each individual ingredient of a food has a unique absorption spectrum. Typically, water has a distinctly higher level of absorption in the IR region compared with that in the visible light region. Multispectral analysis has also been used to quantify various food components such as oil, water [22], vinegar [25], soluble protein [35], and sugar [26,27,28,29,30,31,32,33,34]. Such methods, however, required spectrometers, light sources, and hyperspectral image acquisition equipment, which led to problems such as cost, size, and power consumption, making it difficult to implement on wearable devices.

Multispectral imaging is a method of acquiring individual images from a single wavelength light source, including the UV and near-infrared (NIR) regions, and using them to analyze food [29,37]. This method does not require equipment such as a spectrophotometer and is easy to implement using an optical camera with several LEDs and a silicon imaging sensor, which allows an analysis of food using an approximated light absorption distribution. In a study of detecting vinegar and dressing oil on the surface of lettuce leaves using a light source composed of 10 LEDs with different wavelengths, an accuracy of 84.2% was achieved when using five LEDs [25]. Multiwavelength imaging techniques have been widely applied to the detection and quantification of various components in food. (e.g., sugar in sugarcane [27], water in beef [22], sugar in peaches [28], soluble solids in pomegranates [31], sugar in apples [26], sugar in black tea [33], sugar in potatoes [32], and soluble protein in oilseed rape leaves [35]). The calorie content of a food can be calculated from the estimated amount of each ingredient.

In this study, we applied multiwavelength imaging techniques to the task of categorizing foods and estimating their caloric content. The feasibility of using NIR/UV images for food classification and caloric estimation was verified in terms of accuracy. This was especially true for similar looking foods. Despite the many advantages of multi-wavelength imaging techniques, several issues remain that should be addressed for practical implementation using small wearable devices. Since the number of wavelengths is related to the resolution of the absorption/reflective spectrum of a multiwavelength image, accomplishing accuracy in food analysis makes it highly preferable to use as large a number of wavelengths as possible. Using many wavelengths, however, increases the problems associated with light source control along with drive circuitry, which leads to problems such as large bulk, high power consumption, and heat generation. Another issue is the increased time required for food analysis. Even if the analysis itself is completed in a very short time, the total acquisition time of the image (before analysis) increases linearly with the number of wavelengths applied. When the above issues are considered, choosing a smaller number of wavelengths is preferable from the perspective of practical implementation.

In the present study, we focused on ways to mitigate the challenges caused by acquiring multi-wavelength images while retaining the advantages of multi-wavelength imaging. Previous work has investigated the use of RGB images to estimate other types of images, such as depth distribution [38,39] and semantic segmentation map [40]. An attempt has also been made to use RGB images to predict IR images for the visualization of vegetation map [41,42] and vein patterns [43]. In the present study, we proposed a method that uses image conversion techniques to produce single-wavelength predictions of UV/NIR images from RGB images to accomplish food classification and caloric estimation. Before recommending such a method, there was a clear need to guarantee that the single-wavelength images estimated from the RGB image would approximate the actual images. Therefore, we evaluated the performance of image conversion for a variety of everyday foods with respect to the accuracy of food classification and caloric estimation.

## 2. Materials and Methods

### 2.1. Data Acquisition

The types of foods used in this study are listed in Table 1, along with their calorie counts. The foods used in the experiment were selected from everyday foods, taking into account various physical properties and health effects. Calorie counts for each food were calculated using the calorie-per-weight value and measured weight, if Nutrition Facts are available. For foods without nutrition facts, calorie values were calculated using various nutritional information (food composition, calorie-per-weight, cooking method, etc) published by the Korea Food and Drug Administration (KFDA) [44] and measured weights. Actual measured caloric values were accomplished without using the food analysis equipment in this study, because unevenly distributed food ingredients could lead to different values depending on the sample location. Moreover, the objective was to estimate representative caloric counts using visual cues, and it was reasonable to use values measured by a recognized organization. All liquid foods were captured by placing the same amount of food in the same cup. This was done to suppress the adverse effects that could be caused by different cup shapes and volumes on food classification and caloric estimation. There were many food pairs that were visually similar but nutritionally different, such as (coffee, coffee with sugar), (cider, water), (tofu, milk pudding), (milk soda, milk).The usefulness of UV and NIR images for food classification and caloric estimation was effectively demonstrated by the selection of these foods.

**Figure 1:**
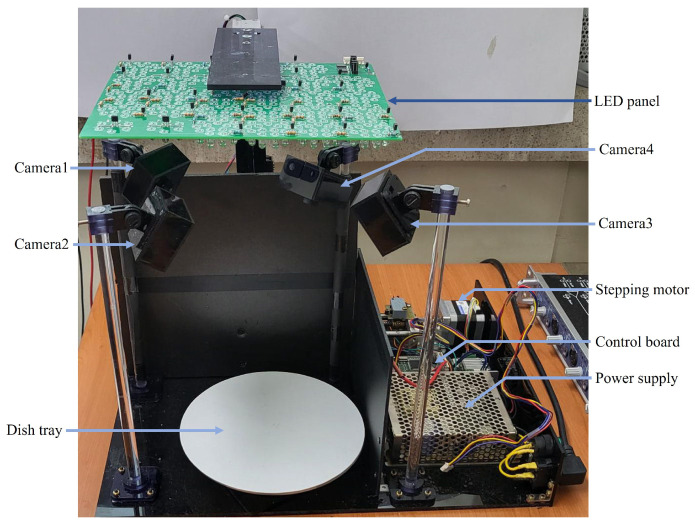
Photograph of the image acquisition system.

The acquisition of multispectral images was achieved by using a custom-made image acquisition system shown in Figure 1. The food was placed on a tray and the distance between the tray and the light source was approximately 25 cm. Four digital cameras (Arducam 1080p, Nanjing, China) were used, each facing the center of the food tray, with an acquired image size of 640 × 480 pixels (HV). The camera was equipped with a CMOS imaging sensor (Omnivision OV2710, Santa Clara, CA, USA) had a field of view of 100° × 138° (HD). The depth of each pixel was represented at 16 bits.

Each camera was equipped with an IR cut filter that was activated by an external control signal. By using this, visible light was blocked when capturing NIR images. The light source was made up of a total of 20 LEDs (Marubeni Φ5 through hole-type, Chiyoda-ku, Tokyo, Japan) emitting different wavelengths of light (385, 405, 430, 470, 490, 510, 560, 590, 625, 645, 660, 810, 850, 870, 890, 910, 950, 970, 1020 nm, and white). The wavelengths were chosen to ensure that the quantum efficiency of the image sensor in the camera used was at least 10%. The white LEDs were used to acquire the RGB images and the other LEDs were intended to obtain the images of the corresponding wavelengths. Each light source consisted of 10 white LEDs and 30 LEDs for each wavelength. The light source for each wavelength was shaped like a circle with a diameter of 42 mm. This was calculated from the radiation angle of the adopted LEDs and the distance between the light source and the food so that entire area of the food tray was illuminated. The center of the light source at a specific wavelength was shifted to that of the food tray before image acquisition at that wavelength. A linear stage driven by a stepping motor (Sanyo-Denki SF2422-12B41, Toshima-ku, Tokyo, Japan) was used to move the light source.

Data augmentation in previous studies was achieved primarily through artificial image transformation. In this study, however, four separate cameras and a rotating table were employed to obtain real images from as many angles as possible. The angular resolution of the rotary table was adjustable from 1 to 30°. A microcontroller that received control commands from a desktop PC performed all operations of the acquisition system, including moving the LED panel, rotating the table, and turning on and off each LED. Two datasets were prepared for food classification and calorie estimation, respectively. The individual data in the first dataset consisted of image-food item pairs, and the individual data in the second dataset consisted of image-calorie value (presented in Table 1) pairs. For foods without nutrition facts (bread and butter, coffee with sugar, sweet milk, steamed pork, steamed rice, rice cake, rice cake and honey, and salad1 with olive oil), the calorie count was calculated by the following process:

(1) Get the nutritional information for the individual ingredients in the food.
(2) Measure the weight of each ingredient in the food.
(3) Calculate the total calories using the nutritional information and the weight of each ingredient.
(4) Mix the ingredients sufficiently (in the case of mixed foods) and acquire the image.

For foods for which nutrition facts were provided, calories were calculated using only the measured weight and nutrition information for the food.

### 2.2. Food Classification and Caloric Estimation

**Figure 2:**
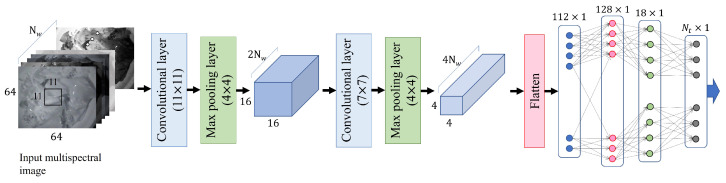
The CNN architecture for food classification, where $N_{w}$ is the number of input images and $N_{t}$ is the number of targets. (101 for food classification and 1 for caloric estimation).

Automatic food classification can be formulated as a general pattern recognition problem based on images. In the multi-wavelength approach, instead of using just one of the RGB images, a combination of images acquired at each wavelength was used to classify the food items or to estimate the specific ingredients or calories in a particular food. Accordingly, two issues should be considered: the design of the pattern recognition methods particularly for food images and the optimal wavelength combinations in terms of food classification/analysis. In the present study, convolutional neural networks (CNNs) were employed to classify food images. A validation dataset was used to heuristically determine the architecture of the CNN (Figure 2). The ratio of the number of images in the validation and learning datasets was 1:10. Since pattern recognition was performed within a single category of food recognition, the resultant CNN had a relatively simple architecture compared with previously developed CNNs (e.g., VGG-16 [14], ResNet152+ANN [19], Inception-v3) that considered a variety of images and categories.

Rather than the full size of an image, it was experimentally confirmed that a reduced image (64 × 64) was more beneficial in terms of classification/estimation accuracy. No cropping was needed to extract the food portion. Accordingly, the steps for classification and estimation could be carried out on the entire image, which included both background and food.

A rectified linear unit (ReLU) was used as the activation function for all of the hidden layers. For the output layer, a soft-max function and a linear combination function were adopted for food classification and caloric estimation, respectively. The loss function for caloric estimation was given by the mean absolute percentage error (MAPE). The MAPE in calories for the *i*-th food item is given by

(1) $$MAPE_{i}=\frac{|M_{i}-{\hat{M}}_{i}|}{M_{i}}$$

where ${\hat{M}}_{i}$ and $M_{i}$ are the estimated and true calories, respectively, for the *i*-th food item. As for food classification, the cross-entropy was adopted as the loss function. As shown in Table 1, some foods have a reference caloric value of zero, in which case the MAPE cannot be calculated. In the present study, a caloric amount of zero was replaced with a minimum value of 5 (kcal), as defined by [44]. When training CNNs for both caloric estimation and food classification, the losses converged when the number of epochs exceeded 1000. The mini-batch size was set to 32, which produced the best performance in all cases.

Although the determination of the optimal wavelength combination was an offline process, training and evaluating a CNN for every combination of wavelengths was very time consuming. In this study, a piecewise selection method [45] was used to reduce the time to determine the optimal combination as opposed to obtaining similar results by using a brute-force grid search. A piecewise selection method is basically an incremental construction method. The set of wavelengths is constructed step by step by adding or removing wavelengths either to or from the previously constructed set.

### 2.3. Prediction of UV/NIR Images Using RGB Images

In food analysis, UV/NIR images have many advantages over optical RGB images from a complementary perspective, but there are some issues that must be addressed from an implementation perspective. Compared with RGB images that can be acquired under natural light, capturing UV/NIR images requires a separate light source that emits light in that specific range of wavelengths. This means a separate space and drive circuit for the UV/NIR light source is required, which is problematic for smaller-sized wearable devices. The camera employed in this study has a quantum efficiency of more than 40% in the 385–1020 nm wavelength band, which eliminates the need for additional UV/NIR cameras. Images for each wavelength cannot be simultaneously acquired, however, and must be gathered in separate acquisition instances. As the number of wavelengths increases, image acquisition time increases, which can lead to issues such as camera displacement effects during acquisition, as well as to long periods of time needed for analysis. This means that achieving high performance in food analysis comes at the cost of increased hardware complexity and longer analysis times.

If it is possible to obtain UV/NIR images from RGB images, the benefits of UV/NIR imaging for food analysis could be realized with much less time for acquisition and with no changes in hardware. Previous studies have demonstrated the feasibility of using RGB images to predict a different domain for its application-specific representation [41]. This study aims to improve the accuracy of food classification and caloric estimation using UV/NIR images predicted from RGB images instead of captured (real) UV/NIR images by using the image conversion techniques.

**Figure 3:**
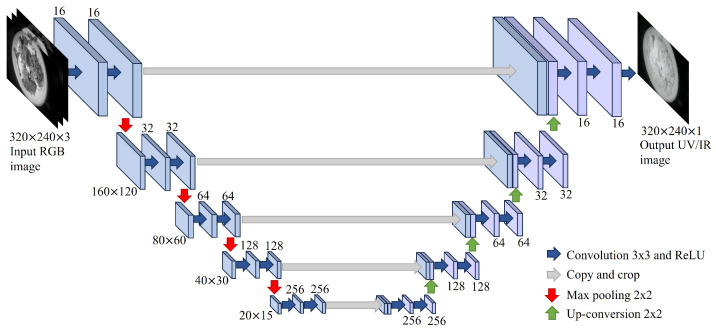
The architecture of the CNN that was used to convert RGB images to UV/NIR images.

Using RGB images to estimate UV/NIR images could be basically be formulated as a problem of finding the pixel-by-pixel mapping rules between the two images. This is based on the assumption that a large amount of low-level information, such as the location of edges, is shared between the two images [43]. Despite the existence of shared information between the two images, each image has unique characteristics that cannot be explained by a simple dependency relationship. Therefore, it was reasonable that the correspondence between the two images was represented by non-linear mapping rules such as those of deep neural networks [38,40,41,42,43,46]. Similarly, a CNN was adopted to estimate UV/NIR images from RGB images in the present study. The CNN architecture used in this study is shown in Figure 3, which basically is similar to that of U-net [46]. In a previous study, a dual encoder-decoder based architecture with different depths [41] and conditional generative adversarial networks [42,43] was employed to estimate NIR images from optical RGB images. These two architectures were tested in terms of food recognition and caloric estimation accuracy on the image dataset used in the present study. No clear performance advantage over the structure shown in Figure 3 was observed in our experiments. A small modification was made to the architecture of the original U-net to meet the objectives of the present study (food classification and caloric estimation). At each layer, the convolution kernel size (3 × 3), image depth (3-16-32-64-128-256-256-128-64-32-16-1) and pooling type (2 × 2 max pooling) was determined empirically using the validation dataset.

A backpropagation algorithm using the Minimum Mean Square Error (MMSE) Square Error (MMSE) criterion was used to train the CNN. The objective function is given by the mean square error between the estimated and the actual UV (or NIR) images, as follows:

(2) $$E=\frac{1}{N}\sum_{n=1}^{N}{\left\{F\left(W,X_{n}\right)-Y_{n}\right\}}^{2}$$

where $F(W,X_{n})$ is the output of the CNN with a set of kernels W where the input RGB image $X_{n}$ is given. $Y_{n}$ denotes the target image (UV or NIR image) at frame index *n* and *N* is the total number of training images. In order to improve the learning convergence, a stochastic gradient descent algorithm was performed in mini-batches with multiple epochs. The updated estimate of the set of kernels W with a learning rate λ is iteratively calculated as follows:

(3) $$W_{n+1}=W_{n}-\lambda {▽}_{W}E$$

For image-to-image conversion, there are several metrics that could be employed to evaluate the performance of a trained neural networks. In this study, however, performance should be evaluated in terms of caloric estimation and food classification accuracy rather than how visually similar the estimated images are to the actual image. To this end, each metric was quantitatively analyzed for estimation accuracy to determine which had more significantly appraised the performance. The results are presented in the following experimental results section.

## 3. Experimental Results

### 3.1. Image Conversion

We first evaluated the performance of the image conversion (RGB-to-UV and RGB-to-NIR). A total of 10,908 pairs of RGB-(UV/NIR) images were used to train the CNN for image conversion, and 3636 RGB images were evaluated separately. The wavelengths targeted for conversion were 385, 405, 810, 850, 870, 890, 910, 950, 970 and 1020 nm, with an equal number of images in each wavelength. Objective measures used to evaluate the conversion performance included peak signal-to-noise ratio (PSNR) and structural similarity index mapping (SSIM) [47]. The results appear in Table 2. The PSNR showed values that approximated 30 dB for all wavelengths except for 385 nm. The highest PSNR of 34.28 dB was observed at the 385 nm wavelength, which correspondingly had the lowest error. When similar values for SNR were observed at all wavelengths, this was due mainly to the fact that the morphological characteristics of similar food items were not changed, and only the brightness values within each boundary of the image were affected. In applications such as image compression, if the PSNR of the restored image is close to 30 dB, the corresponding image is visually similar to the original image without unnoticeable distortion. Thus, the experimental results indicate that the image at each wavelength predicted by RGB could serve as a substitute for the actual acquired image from a visual perspective. The SSIM values for each wavelength also showed no significant deviation from the overall average. However, the SSIM showed behavior that differed slightly from that of the PSNR. The maximum SSIM was obtained at 810 nm. The SSIM was lowest at 405 nm, but the PSNR was relatively high (31.05 dB) at that wavelength. Although the target image was different, the results are generally similar to previous RGB-to-NIR image conversion techniques (e.g., SSIM value of 0.847 at 820 nm [42]).

**Figure 4:**
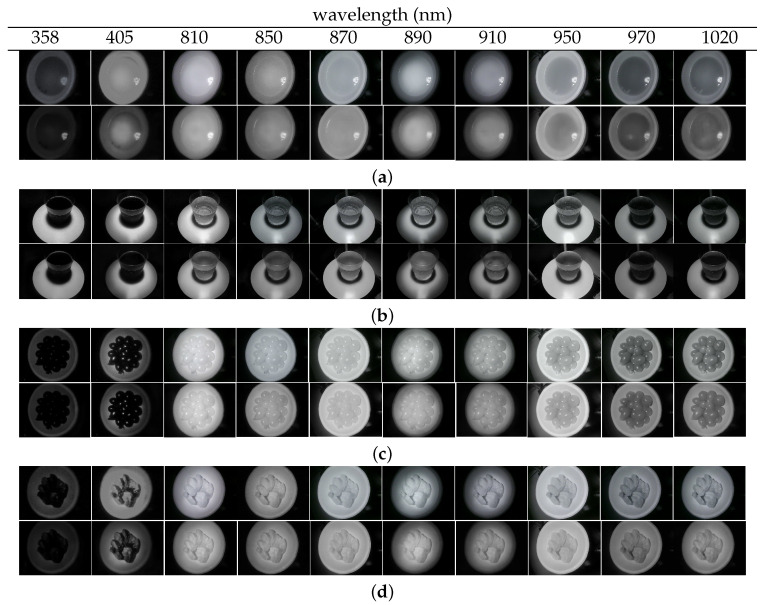
Examples of the original (top)/predicted (bottom) images at each wavelength for (**a**) corn soup, (**b**) coke, (**c**) tomato, and (**d**) pork.

Examples of the UV/NIR images predicted from RGB images appear in Figure 4, along with the actual captured images for comparison. As shown in the figure, the predicted images are in close visual agreement with the actual images acquired by the camera. These results were somewhat expected based on the objective metrics. However, some spots in areas of uniform brightness (e.g., the coke region in the “coke image”) were occasionally found in the predicted image.

### 3.2. Food Classification

The correct classification rates for food images according to the number of wavelengths when using actual images from the camera appear in Table 3. For comparison, the four different NN architectures using RGB images alone were tested, which included VGG-16 nets [14], ResNet152+ANN [19], and a wide hierarchical subnetwork-based neural network (WI-HSNN) [20]. The output nodes of these neural networks were adjusted to match the number of food items adopted in this study, and the classification accuracy was compared with the proposed NN architecture. All images, except for the RGB images, were acquired from UV and NIR light sources.

The four neural networks using only RGB images showed similar classification accuracy, as shown in Table 3. The proposed neural network architecture revealed only a 0.81% difference in classification accuracy compared to the WI-HSNN, which showed the highest accuracy. The results showed that the addition of just a single wavelength image at 970 nm to the RGB image increased the recognition rate by 10.32%. This was due primarily to a significant increase in recognition rates for food pairs that looked very similar but had differences between the UV or NIR images [45]. The highest recognition rate was 99.45% when recognition was performed using eight single-wavelength images in addition to the RGB image. When all wavelengths of images (11 including the RGB image) were used, the recognition rate was slightly lower than its maximum, which was likely a result of overtraining due to excessive image usage. The correlation coefficient between the recognition rate and the number of wavelengths was 0.767, which indicated a significant increase in the recognition rate with the number of wavelengths. However, this also indicated that increasing the recognition rate comes at a cost: more lights, more image acquisition time, etc.

As a way to solve this problem, the results of food recognition obtained by using the predicted UV/NIR images from RGB images are presented in Table 4. The average PSNRs and SSIMs are also presented as prediction performance metrics for images at each selected wavelength. As with using the actual captured image, adding the predicted single-wavelength image improved the classification rate by 5% over using the RGB image alone. The maximum accuracy was obtained when all single wavelength images were combined with the RGB image to train the neural network for food classification, with a value of 98.24%. It is noteworthy that in this case, all single-wavelength images were obtained from RGB images, so there was no need to increase either the acquisition time or the number of wavelengths (or, equivalently, the number of light sources) as when using actual images. The selected wavelengths were different from when actual images were used and the classification rates were slightly lower than when using actual images. The difference in the maximum classification rate between the two cases (using actual or predicted images) was only 1.21%. Using the actual images, however, would require a total of nine image acquisitions, which implies nine different LED light sources and a nine-folds increase in acquisition time.

The relationships between each of the image conversion metrics and the recognition rates were also analyzed. There was a positive correlation between the PSNR and the classification rate, with a value of 0.340, which is insignificant. The correlation coefficient between SSIM and the recognition rate was −0.522, which means that even if the predicted image approximates the actual image in terms of SSIM metrics, the recognition rate could be worse. These results suggest that the metrics employed for image prediction are not significantly related to recognition rate.

The experiment was also conducted in which the conversion rules (RGB-to-UV, RGB-to-NIR) from the images acquired in this study were applied to the images in the well-known food image dataset, such as FOOD-101. Since the reference images (single wavelength images) were not available in FOOD-101, it was impossible to evaluate the performance of image conversion in terms of PSNR, SSIM, etc. However, meaningful performance improvements were achieved, when food classification rules were constructed using the estimated UV/NIR images. This indicates that although the conversion rules were not built from the FOOD-101 dataset, these conversion rules were useful for multi-wavelength food classification for the FOOD-101 dataset.

### 3.3. Caloric Estimation

The results of estimating calories from images of food appear in Table 5 when training a neural network using RGB images alone and UV/NIR images together. In the case of using RGB image alone, the VGG-16 nets with the linear activation function at the output node [14] was also tested for comparison. The two neural networks using only RGB images reveled similar performance in terms of MAPE (28.65 vs. 27.95). The MAPE was decreased by 24.12% (from 28.65 to 21.74) when one NIR image at 970nm was used with an RGB image. This was the maximum reduction that could be achieved by increasing the number of wavelengths by one, which indicates that the addition of only a single wavelength image to an RGB image could result in the greatest reduction in MAPE.

While the MAPE values decreased as the number of wavelengths increased, there was a significant increase in MAPE values when using images of all wavelengths adopted in this study. This appears to be a side effect of using too many multiwavelength images, as evidenced by the fact that the MAPE value actually increases as the number of images increases from 8 to 9. Excluding the maximum number of wavelengths (11), the correlation coefficient between MAPE and the number of wavelengths is −0.8471, indicating that MAPE decreases significantly with the number of wavelengths. Linear regression analysis also showed that the MAPE was decreased by 1.034 when the number of wavelengths was increased by one. The minimum MAPE (11.67) was obtained when a total of 8 images was used, which includes RGB images. Similar to food classification, it is apparent that an 8-fold acquisition of images is required compared with the conventional method using only RGB images.

So far, the results were obtained by using the actually captured images. The results of caloric estimation using UV/NIR images predicted from RGB images are presented in Table 6. The results are similar to using actual acquired images. When the neural network was trained by adding just one type of single-wavelength NIR image to the RGB image, a 37.55% reduction in MAPE was achieved. The first wavelength selected was 970 nm, which approximates the 950 nm that was observed when using the actual images. The correlation coefficient between MAPE and the number of images (number of wavelengths) was −0.7166, which is slightly lower than when using actual acquisition images. The lowest MAPE value was obtained when a total of 11 images was used, indicating that more images were needed when using predicted images compared with using actual images, such as for food classification. The difference in the minimum MAPE value between using the actual acquisition images and using the estimated images was only 0.46, which was is not a significant difference. Such results demonstrate that image conversion techniques are useful in caloric estimation with high accuracy while overcoming the challenges associated with an increased number of light sources and repeated image acquisition.

A correlation analysis between the accuracy of caloric estimation and the objective metrics of image conversion was also investigated. Both PSNR and SSIM have negative correlation coefficients, which means that a better conversion performance equates to a more accurate caloric estimation. However, the absolute value of the correlation coefficient is very small (0.097 and 0.256 for PSNR and SSIM, respectively), and indicates that PSNR and SSIM, which we used as metrics of conversion performance in this study, do not significantly affect the accuracy of caloric estimation. These results suggest that, as in food classification, the metrics in image conversion that are more closely related to the accuracy of caloric estimation should be explored.

## 4. Conclusions

Image-based food analysis technology is an attractive method since it does not require expensive specialized equipment and can be implemented on existing wearable devices. It is essential that the precision of image-based analysis is at least comparable to what could be achieved with specialized equipment. To this end, multi-wavelength image analysis was adopted in which multiple images acquired from multiple narrow-band wavelength light sources including UV and NIR lights were used. Such an approach showed significantly higher accuracy in food classification and caloric estimation compared to using RGB images only. There are the drawbacks, however, of requiring multiple light sources and long acquisition times. To mitigate these problems, we propose the use of converted RGB images instead of actual UV/NIR images acquired using a camera.

It was experimentally confirmed that the UV/NIR images estimated from the RGB images were very similar to the originals from both visual and objective perspectives. The performance of the multi-wavelength food analysis techniques using the estimated images approximated the use of actual images in terms of both food classification and caloric estimation. In conclusion, high performance multi-wavelength imaging techniques could be achieved using conventional RGB images with only a software change. As future study, we will focus on image conversion techniques that improve not only the visual and objective similarities between the converted and original images, but also on the precision of food analysis.

## Figures and Tables

**Table 1:** Dataset properties per food item.

Food Name	Caloric Count (kcal)	Food Name	Calorie Count (kcal)
apple juice	N/a	pork (steamed)	441.41
almond milk	41.57	potato chips	130.82
banana	127.80	potato chips (onion flavor)	133.95
banana milk	110.27	sports drink (blue)	17.00
chocolate bar (high protein)	167.00	chocolate bar (with fruits)	170.00
beef steak	319.39	milk pudding	189.41
beef steak with source	330.29	ramen (Korean-style noodles)	280.00
black noodles	170.00	rice (steamed)	258.45
black noodles with oil	N/a	rice cake	262.46
blacktea	52.68	rice cake and honey	288.60
bread	129.54	rice juice	106.21
bread and butter	182.04	rice (steamed, low-calorie)	171.18
castela	287.68	multi-grain rice	258.08
cherryade	79.06	rice noodles	140.00
chicken breast	109.00	cracker	217.88
chicken noodles	255.00	salad1 (lettuce and cucumber)	24.20
black chocolate	222.04	salad1 with olive oil	37.69
milk chocolate	228.43	salad2 (cabbage and carrot)	17.28
chocolate milk	122.62	salad2 with fruit-dressing	28.04
cider	70.55	armond cereal (served with milk)	217.36
clam chowder	90.00	corn cereal (served with milk)	205.19
coffee	18.56	soybean milk	85.95
coffee with sugar (10%)	55.74	spagetti	373.73
coffee with sugar (20%)	92.92	kiwi soda (sugar-free)	2.34
coffee with sugar (30%)	130.11	tofu	62.37
coke	76.36	cherry tomato	36.00
corn milk	97.18	tomato juice	59.80
corn soup	85.00	cherry tomato and syrup	61.90
cup noodle	120.00	fruit soda	27.04
rice with tuna and pepper	418.15	vinegar	20.16
dietcoke	0.00	pure water	0.00
choclate bar	249.00	watermelon juice	79.97
roasted duck	360.98	grape soda	92.43
orange soda	33.33	grape soda (sugar-free)	0.00
orange soda (sugar-free)	2.77	fried potato	331.50
fried potato and powder	364.92	yogurt	114.56
sports drink	47.23	yogurt and sugar	106.04
ginger tea	96.79	milk soda	86.84
honey tea	126.69	salt crackers	218.89
caffelatte	79.13	onion soap	83.00
caffelatte with sugar (10%)	115.66	orange juice	82.17
caffelatte with sugar (20%)	152.19	peach (cutted)	55.38
caffelatte with sugar (30%)	188.72	pear juice	90.02
mango candy	91.00	peach and syrup	124.80
mango jelly	212.43	peanuts	217.96
milk	94.50	peanuts and salt	218.21
sweet milk	N/a	milk tea	63.46
green soda	84.55	pizza (beef)	212.08
pizza (seafood)	148.83	pizza (potato)	179.34
pizza (combination)	175.87	plain yogurt	109.89
sports drink (white)	43.95		
		mean	139.27
		standard deviation	101.36

**Table 2:** Image prediction performance.

Wavelength (nm)	385	405	810	850	870	890	910	950	970	1020	Avg.	Std.
PSNR (dB)	34.28	31.05	30.18	29.71	30.15	30.76	30.63	29.38	30.06	29.87	30.61	1.38
SSIM	0.863	0.774	0.912	0.871	0.906	0.906	0.831	0.876	0.856	0.851	0.865	0.042

**Table 3:** Food classification accuracies for each of selected wavelengths, in the case of using actual captured images.

No. of Images	Selected Wavelengths (nm)											Acc. (%)
1	RGB	(VGG16) [14]										85.54
1	RGB	(ResNet152+ANN) [19]										87.23
1	RGB	(WI-HSNN) [20]										88.04
1	RGB	(proposed)										86.30
2	RGB	970										96.62
3	RGB	910	970									98.60
4	RGB	405	910	970								98.43
5	RGB	405	910	950	970							98.71
6	RGB	385	405	910	950	970						99.06
7	RGB	385	405	890	910	950	970					99.23
8	RGB	385	810	850	890	910	950	970				99.37
9	RGB	385	810	850	870	890	910	950	970			99.45
10	RGB	385	810	850	870	890	910	950	970	1020		99.06
11	RGB	385	405	810	850	870	890	910	950	970	1020	99.15

**Table 4:** Food classification accuracies, average PSNR, and average SSIM for each of selected wavelengths for food classification, in the case of using predicted images.

No. of Images	Selected Wavelengths (nm)											Acc. (%)	Avg. PSNR	Avg. SSIM
1	RGB											90.23	–	–
2	RGB	950										95.24	29.38	0.876
3	RGB	385	870									96.89	32.22	0.885
4	RGB	385	870	1020								97.61	31.43	0.874
5	RGB	385	810	870	1020							96.59	31.12	0.883
6	RGB	385	810	850	870	970						98.13	30.88	0.882
7	RGB	385	405	810	850	870	1020					97.91	30.87	0.863
8	RGB	385	405	810	850	870	910	1020				97.85	30.84	0.858
9	RGB	385	405	810	850	870	910	970	1020			98.16	30.74	0.858
10	RGB	385	405	810	850	870	890	910	950	970		97.77	30.69	0.866
11	RGB	385	405	810	850	870	890	910	950	970	1020	98.24	30.61	0.865

**Table 5:** Caloric estimation results for each of selected wavelengths, in the case of using actual captured images.

No. of Images	Selected Wavelengths (nm)											MAPE
1	RGB	(VGG16) [14]										27.95
1	RGB	(proposed)										28.65
2	RGB	970										21.74
3	RGB	385	1020									18.54
4	RGB	385	970	1020								18.30
5	RGB	385	850	970	1020							14.57
6	RGB	385	850	890	970	1020						14.29
7	RGB	385	405	850	910	979	1020					12.63
8	RGB	385	405	850	910	950	970	1020				11.67
9	RGB	385	405	850	870	910	950	970	1020			15.00
10	RGB	385	405	810	850	870	910	950	970	1020		12.68
11	RGB	385	405	810	850	870	890	910	950	970	1020	21.42

**Table 6:** Caloric estimation results for each of selected wavelengths, in the case of using predicted images.

No. of Images	Selected Wavelengths (nm)											MAPE	Avg. PSNR	Avg. SSIM
1	RGB											32.28	–	–
2	RGB	970										20.16	30.06	0.856
3	RGB	385	970									17.59	32.17	0.859
4	RGB	385	405	970								16.65	31.80	0.831
5	RGB	385	405	850	970							18.19	31.28	0.841
6	RGB	385	405	850	890	970						15.96	31.17	0.854
7	RGB	385	850	870	890	970	1020					16.21	30.81	0.876
8	RGB	385	405	850	870	890	970	1020				17.05	30.84	0.861
9	RGB	385	405	850	870	890	950	970	1020			17.41	30.66	0.863
10	RGB	385	405	810	850	870	890	910	950	1020		12.13	30.60	0.868
11	RGB	385	405	810	850	870	890	910	950	970	1020	18.71	30.61	0.865

## Data Availability

The data presented in this study are available on request from the corresponding author.
